# The Book World of Medicine and Science

**Published:** 1911-05-27

**Authors:** 


### THE BOOK WORLD OF MEDICINE AND SCIENCE.

Ambulance Examination Questions, being a Catechism on First-Aid to the Injured and Sick. By D. M. Macdonald, M.D., M.R.C.P.E. (Bristol: John Wright and Sons, Ltd. 1911. Price 6d. net.)

This booklet has met with such success that a fourth edition has been called for. The scope of the questions set forth in it is comprehensive and such as would tax the knowledge of even an advanced first-aid pupil. It is perhaps inadvisable to aim high when determining what questions to ask candidates for ambulance certificates, since in matters medical a little knowledge may be more dangerous than complete ignorance. Nevertheless, at times the author seems to expect a good deal from his pupils. Thus in one place he asks, "What do you know of nepenthe?" and in another, "What treatment would you follow in poisoning by pearlash?" Here and there again questions are loosely put, *e.g.* under the heading emergencies, he asks, "How would you render aid to, and what treatment would you apply in the following cases, pending arrival of medical aid: II. "A fire in the eye?" or again, under "Problems": I. "How would you remove a leech from the stomach swallowed by mistake?" Presumably it was the leech not the stomach that was swallowed! A clear account of the Schäfer method for restoring animation in the apparently drowned is given in an appendix. On the whole it may be said, however, that anyone who can answer the author's questions correctly ought to be considered capable of dealing with any ordinary emergency.

Lepers: Thirty-six Years' Work among Them. By John Jackson, F.R.G.S. (London: Marshall Bros., Limited, and The Mission to Lepers. 1910.

This history of the Mission to Lepers is intended to crystallise into actual knowledge the vague ideas that are held by very many persons with regard to lepers. The reader is brought in touch with the devotion of those men and women who give their lives to the work amongst lepers. A very important branch is carried on for the rescue of the children of leper parents and we learn that in the twenty-two homes supported by the Mission five hundred boys and girls are being saved from the loathsome disease and from a life of misery. The story unfolded at length in this book is intensely graphic and gives a vivid picture of the truly awful social condition of these unfortunate people. The perusal of this record cannot fail to move those interested, and to promote the extension of such a merciful work—a work which has escaped those criticisms so frequently made in regard to many of the missions sent out to convert the "heathen."

